# Validity and reliability of the Box and Block Test for individuals with schizophrenia spectrum disorder

**DOI:** 10.3389/fpsyt.2025.1584352

**Published:** 2025-06-06

**Authors:** Jing-Wen Su, Hsiang-Yu Chen, Kuan-Yi Li

**Affiliations:** ^1^ Department of Occupational Therapy and Graduate Institute of Behavioral Sciences, Chang Gung University, Taoyuan, Taiwan; ^2^ KangHsin Psychiatric Halfway House, Taoyuan, Taiwan; ^3^ YangFeng Psychiatric Community Rehabilitation Center, Taoyuan, Taiwan; ^4^ Division of Movement Disorders, Department of Neurology, Chang Gung Memorial Hospital at Linkou, Taoyuan, Taiwan; ^5^ Healthy Aging Research Center, Chang Gung University, Taoyuan, Taiwan

**Keywords:** dexterity, mental illness, Minnesota Rate of Manipulation Test, hand function, Minnesota Manual Dexterity Test, Purdue Pegboard Test, Box and Block Test

## Abstract

**Background:**

The Box and Block Test (BBT) is recognized for assessing manual dexterity; however, its reliability and validity, specifically for individuals with schizophrenia spectrum disorders, remain underexplored. The objective of this study was to establish the validity and reliability of the BBT for individuals with schizophrenia spectrum disorders.

**Methods:**

This cross-sectional observational study was conducted in community psychiatric rehabilitation centers. The participants were individuals with schizophrenia spectrum disorders, ranging in age from 20 to 65 years. A total of seventy participants underwent the BBT, Purdue Pegboard Test (PPT), and Minnesota Manual Dexterity Test (MMDT) to assess manual dexterity. The validity and reliability of the BBT were evaluated using Pearson correlation coefficient and intraclass correlation coefficient (ICC).

**Results:**

The BBT strongly correlated with the PPT across all subtests (p < 0.001) and demonstrated moderate to strong correlations with the MMDT, except for the two-hand turning subtest. Intra- and inter-rater reliability ICCs ranged from 0.80 to 0.95 (p < 0.05), and test-retest reliability ICCs were between 0.70 and 0.71 (p < 0.001). A notable moderate negative correlation was observed between manual dexterity performance and both the total score and the positive symptom subscale of the Brief Psychiatric Rating Scale-Chinese version (BPRS-C) (r = -0.42 ~ -0.56, all p <0.001). Additionally, there were significant low correlations between the BBT and monthly income (r = 0.29 ~ 0.30, all p = 0.01).

**Conclusions:**

BBT has good validity and reliability in individuals with schizophrenia. Thus, BBT has emerged as a more favorable option for clinical assessment, avoiding the limitations that hamper PPT and MMDT. The simplicity and rapidity of BBT, combined with the provision of normative data, support the creation of customized rehabilitation plans that are crucial for rehabilitation focused on vocational and daily living skills.

## 1 Introduction

Schizophrenia, a complex mental disorder, involves genetic and environmental factors (1). It impairs cognition and motor functions, presents with positive and negative symptoms, and displays catatonic features, such as motor signs, extrapyramidal symptoms, and psychomotor slowing (2, 3), leading to tremors, stereotypies, and coordination issues. Motor dysfunctions hinder daily activities and are observed in various body parts, affecting postural control, eye and limb movements, and fine motor skills (4, 5). Often, antipsychotic medications contribute to motor deficits by inducing extrapyramidal side effects such as dystonia, akathisia, dyskinesia, and Parkinson-like symptoms (4, 6).

Manual dexterity is vital for individuals with schizophrenia in community psychiatric rehabilitation and influences treatment, daily activities (ADL), and vocational rehabilitation (4). Achieving independence in daily tasks enhances a sense of competence and autonomy (7). However, their upper extremity coordination and hand operation are often inferior to those of healthy adults, affecting the quality of daily activities (8). Térémetz et al. (2017) highlighted significant deficits in force tracking, tapping regularity, and the memory of tapping sequences in individuals with schizophrenia, suggesting that manual dexterity could serve as a clinical marker. This aspect of motor function is not only crucial for daily independence but also correlates with executive and social functions, impacting employment status (9, 10).

Based on previous research, manual dexterity is defined here as the ability to manipulate objects using the hands and fingers (11, 12) and is influenced by various factors, including gender, age, and handedness (13). Studies have shown that manual dexterity declines with age due to reduced muscle and skeletal system quality, slower nerve conduction, and decreased hand proprioception, which impact fine motor task performance (14). Handedness affects dexterity, with the dominant hand typically performing better due to its more precise internal limb dynamics and control (15).

Given the significant role of manual dexterity in the functionality and independence of individuals with schizophrenia, it is imperative to select assessment tools with proven reliability and validity. To guide the selection of a dexterity assessment, Yancosek and Howell (2009) recommended the Box and Block Test (BBT), Purdue Pegboard Test (PPT), and Minnesota Rate of Manipulation Test (MRMT) as preferred tools for evaluating manual dexterity, supported by robust evidence of their psychometric properties. The BBT is a widely used tool for assessing gross manual dexterity. Participants are asked to transfer 1-inch blocks from one side of a box to the other as many times as possible within 60 seconds, testing their ability to grasp, move, and release objects (16). The PPT primarily measures fine motor skills and is frequently applied in both clinical practice and research. It includes four subtests that involve inserting small pins into pegboard holes and assembling pins with washers (17). The MRMT and its updated version, the Minnesota Manual Dexterity Test (MMDT), are designed to assess manual dexterity. Although the MRMT is no longer manufactured, both tests include five timed subtests that require participants to turn or place 60 small, round blocks using one or both hands. The MMDT specifically measures basic eye-hand coordination and arm-hand dexterity with larger objects, focusing mainly on gross motor skills (18).

Currently, there is no consensus on the most appropriate dexterity assessment for individuals with schizophrenia. Various tools, such as the finger tapping test and pegboard test, along with novel approaches, have been applied in both clinical and research settings (4, 9, 19, 20). Among the BBT, PPT, and MMDT, studies have demonstrated that the BBT is particularly sensitive in detecting changes in motor performance, showing moderate to large responsiveness in stroke rehabilitation contexts (21, 22). Compared to other dexterity assessments, the BBT has demonstrated good validity, low floor and ceiling effects, and the ability to capture a wide spectrum of upper limb motor impairments, with moderate to strong correlations to other upper limb assessments in diverse populations (23, 24). However, its validity for use in schizophrenia has not yet been established.

The Purdue Pegboard Test (PPT) assesses unilateral, bilateral, and fine motor coordination, offering a more comprehensive evaluation of dexterity (17). However, Lee et al. (2013) reported that in individuals with schizophrenia, most subtests demonstrated a high minimal detectable change (MDC). This suggests that only substantial score differences can be considered true improvements, thereby limiting the test’s sensitivity (25). Additionally, hand dexterity appears to be a relatively stable trait with limited responsiveness to antipsychotic treatment, making meaningful score improvements difficult to achieve in this population and reducing the test’s clinical utility (25). The MMDT is suitable for evaluating workers performing tasks that require rapid handling of uniform tools and materials, making it particularly relevant for assessing individuals with schizophrenia in vocational rehabilitation contexts. However, Surrey et al. (2003) raised concerns about significant score discrepancies between the placing and turning subtests across two test versions. They also noted that the MMDT continues to rely on normative data and instructions from the original 1957 MRMT version (26). These findings highlight the need for updated normative data and further evaluation of the psychometric properties in MMDT (26).

When selecting outcome measures, practicality and ease of use should be weighed alongside factors such as time requirements, equipment, and necessary training. The importance of efficient assessment tools is highlighted by challenges associated with the PPT and MMDT. Although the PPT takes approximately 10 minutes to complete, research recommends administering three trials per subtest to ensure reliable results (17). The MMDT requires substantial space, with boards measuring at least 81 cm by 22 cm when opened, and conducting the test in a standing position may be difficult for individuals of varying heights due to the fixed table height recommendation (27). Moreover, the MMDT can be time-consuming, involving multiple timed tasks with several trials required for each hand. Both the PPT and MMDT can thus become frustrating or overly complex for individuals with schizophrenia. The BBT, known for its application in stroke rehabilitation, offers a promising alternative due to its shorter duration, simpler procedures, portability, and cost-effectiveness. Preliminary feedback from individuals with schizophrenia suggests a positive response to the BBT; however, its reliability and validity for this group still require thorough investigation. This study aimed to evaluate the validity and test-retest reliability of the BBT in individuals with schizophrenia spectrum disorder. Additionally, this study explored the impact of disease-specific factors on manual dexterity.

## 2 Methods

### 2.1 Participants

The study was conducted between March and May 2023. Approval was obtained from the directors of community psychiatric rehabilitation centers to recruit participants at their respective facilities. Researchers visited these institutions to explain the study’s purpose and procedures. The *a priori* sample size calculation was based on a significance level (alpha) of 0.05 and statistical power (beta) of 80%. The recommended minimum sample size for assessing the intra-class correlation coefficient (ICC), accounting for a 20% anticipated dropout rate, was calculated to be 30 participants (28). In addition, we reviewed previous studies on the validity and reliability of BBT, which reported sample sizes ranging from 20 to 628 participants (16, 29–32). Based on these considerations, the final minimum sample size was determined to be at least 30 participants.

Inclusion criteria were as follows: 1) diagnosed with schizophrenia spectrum disorder by a psychiatrist, without substance abuse, intellectual disabilities, or cognitive disorders; 2) aged 20–65 years; 3) capable of communication and following instructions; and 4) residing in the community. Exclusion criteria were: 1) diagnosis of neurological or musculoskeletal disorders affecting hand dexterity; 2) under assistance or adult guardianship; and 3) cognitive impairment, indicated by a Mini-Mental State Examination (MMSE) score below 24. All participants provided informed consent, and the study protocol was approved by the local Institutional Review Board.

### 2.2 Measurements

#### 2.2.1 Box and Block Test

The Box and Block Test (BBT) comprises assessments for both the dominant and non-dominant hands, following the standardized protocol outlined by Mathiowetz et al. (1985) (16). An 18.7 cm divider divides the BBT box into two sections, with the test compartment housing 150 one-inch cubes. Participants were positioned facing the administrator to ensure easy access to the divider and the ability to pick up a single cube at a time. Prior to formal testing, participants underwent a 15-second practice session. They were then instructed to transfer the cubes from the test compartment to the opposite side as swiftly as possible. Each subtest, conducted for one minute during both the initial and follow-up sessions, assessed test-retest reliability. The validity and reliability of the BBT are well-established, with previous studies by Platz et al. (2005) confirming its high inter-rater reliability and robust construct validity (30).

#### 2.2.2 Purdue Pegboard Test

The Purdue Pegboard Test (PPT) is widely utilized by clinicians and researchers to evaluate gross movements of the arms, hands, fingers, and fingertip dexterity (11). Its psychometric properties have been well-established (17), including test-retest reliability and minimal detectable change (MDC) for individuals with schizophrenia (25). In our study, the PPT was employed as the benchmark for assessing the validity of the Box and Block Test (BBT). The PPT consists of five subtests: right hand, left hand, both hands, right+left+both hands, and assembly tests. However, the “right+left+both hands” score is not an independent test; it represents the combined score of the individual right hand, left hand, and both hand tests (33). The assessment tool is a board measuring 44.4 cm in length, 29.6 cm in width, and 1.8 cm in height, featuring two rows of holes with 25 holes in each row, and four cups on the top. The components include 50 pegs, 40 washers, and 20 collars (17). During the right-hand and left-hand tests (30 s), participants used one hand to insert pegs into the holes. In the two-hand test, also timed for 30 seconds, participants simultaneously use both hands to place pegs from top to bottom into the holes, with scores based on the number of pegs inserted. In the assembly test, timed for sixty seconds, participants are instructed to alternately use both hands to assemble the components in the sequence of ‘peg, washer, collar, washer.’ Throughout the testing, participants are encouraged to perform as quickly as possible.

#### 2.2.3 Minnesota Manual Dexterity Test

The Minnesota Manual Dexterity Test (MMDT) evaluates quick and simple eye-hand coordination as well as arm-hand dexterity (Lafayette Instrument, 1998). Its applications span various fields, including Physical Therapy, Occupational Therapy, vocational assessments, and pre-employment screenings (Lafayette instrument, 1998). Psychometric properties of the MMDT have been established (18). In our study, it served as the benchmark for validating the Box and Block Test (BBT). The MMDT comprises five subtests: placing, turning, displacement, one-hand turning and placing, and two-hand turning and placing (Lafayette Instrument, 1998). The test kit includes a folding plastic board measuring 85.4 cm in length, 22.8 cm in width, and 0.5 cm in height, equipped with 60 holes, accompanied by 60 cylindrical blocks. Test administration followed the procedures outlined in the Lafayette Instruments manual (1998). The guidelines stipulate that the testing table should be between 71.12 cm and 81.28 cm high, with the plastic board positioned 2.54 cm from the table’s edge. Participants were instructed to remain standing and allowed one practice attempt. Each subtest was performed twice, and the administrator recorded the total time required.

#### 2.2.4 Brief Psychiatric Rating Scale-Chinese version

The BPRS-C employs a 7-point Likert scale to gauge symptom severity, with scores ranging from 1 (absent) to 7 (highly severe) for each item. Assessments were conducted through interviews and observations. The total score ranges from 16 to 112 points, with higher scores indicating more severe psychiatric symptoms (34). Additionally, Shafer et al. (2017) identified four factors in this scale: affective syndrome (BRRS-C-affective), positive syndrome (BRRS-C-positive), negative syndrome (BRRS-C-negative), and activation (BRRS-C-activation) (35). Before formal evaluation, interrater reliability was ensured between a psychiatrist with 15 years of experience and the research team.

### 2.3 Procedure

The participants underwent screening using the Mini-Mental State Examination (MMSE) to ensure their cognitive function met the inclusion criteria. Subsequently, interviews were conducted to collect demographic information, and assessments were performed using the BPRS-C. Hand dominance was determined using the Edinburgh Handedness Inventory (36). The Box and Block Test (BBT) was conducted across two sessions spaced two weeks apart. At the same time, the Purdue Pegboard Test (PPT) and Minnesota Manual Dexterity Test (MMDT) were administered only during the initial session. Two experimenters administered the manual dexterity tests. Before data collection, both experimenters extensively reviewed the testing manuals and underwent multiple practice sessions under the supervision of an occupational therapist with 20 years of experience to ensure reliability. The reliability of the raters was confirmed. The ICCs for the intra-rater reliability were 0.95 (CI: 0.57~0.99; p = 0.002) and the ICCs for inter-rater reliability were 0.83~0.93 (CI: 0.36~0.99; p = 0.01). All interviews and assessments were conducted in a quiet, private room, with the entire data collection process taking approximately 60–70 minutes to complete.

### 2.4 Data analysis

Data analysis was conducted using IBM SPSS Statistics for Windows, version 22.0 (IBM Corp. Released 2017. IBM SPSS Statistics for Windows. Armonk, NY: IBM Corp.). Descriptive statistics were used to present demographic data. The validity and test-retest reliability were assessed using Pearson and intraclass correlation coefficients (ICC), respectively. Correlations below 0.3 were classified as small, those ranging from 0.3 to 0.6 were considered moderate, and correlations above 0.6 were deemed strong (37). Statistical significance was set at *p* < 0.05.

## 3 Results

### 3.1 Demographic characteristics

A total of 70 individuals with schizophrenia spectrum disorder participated in the study, including 34 women, with a mean age of 47.24 ± 10.36 years, ranging from 25 to 65 years old. The group exhibited generally preserved cognitive function, with an average Mini-Mental State Examination (MMSE) score of 27.50 ± 2.31. The majority were right-handed, with only five being left-handed. A significant proportion (70%) of the participants had never been married, 80% resided in residential psychiatric rehabilitation facilities, and approximately 48.60% were engaged in a vocational rehabilitation program within these settings. On average, participants had completed 12.01 ± 2.68 years of education.

Concerning disease-related factors, the average disease duration was 20.20 ± 10.75 years, and the BPRS-C score was 12.84 ± 6.03, suggesting mild symptom severity. The mean daily dose of antipsychotics, expressed as chlorpromazine equivalents, was 210.96 ± 332.51 mg/day. Additional demographic details are presented in **Table 1**.

**Table 1:** Demographics characteristics of the participants (N = 70).

Characteristics	Mean (SD) or No. (%)
**Age, years**	47.24 (10.36)
**Types of disease**	
Schizophrenia	59 (84.3%)
Schizoaffective disorder	11 (15.7%)
**Disease duration, years**	20.20 (10.75)
**Monthly income, USD**	146.45 (291.26)
**BPRS-C (Total score)**	12.84 (6.03)
Positive symptoms	3.51 (2.16)
Negative symptoms	4.30 (3.42)
Affection symptoms	3.33 (2.47)
Activity activation symptoms	1.70 (0.94)
**Gender**	
Female (%)	36 (51.4%)
Male (%)	34 (48.6%)
**Education, years**	12.01 (2.68)
**Marital status**	
Never married	49 (70%)
Married	1 (1.4%)
Divorced	15 (21.4%)
Widowed	5 (7.1%)
**Antipsychotics dosage, mg/day**	210.96 (332.51)
**MMSE**	27.50 (2.31)
**Handedness**	
Right	65 (92.9%)
Left	5 (7.1%)

BPRS-C, Chinese version of Brief Psychiatric Rating Scale; MMSE, Mini-Mental Status Examination.

Seventy participants completed the first session, but one participant could not return for the second session to retest the BBT. Therefore, only 69 participants were included in the test-retest analysis.

### 3.2 Manual dexterity performance

The mean score of the dominant hand test in BBT was 54.44 ± 9.64 for males and 56.61 ± 9.98 for females. For the non-dominant hand test, the mean score was 51.15 ± 10.51 for males and 56.61 ± 9.98 for females. Comparing these scores to the norms established for right-handed healthy adults in Taiwan by Li et al. (2020) (38), our participants generally scored below the norms by 0.71-5.66 SD.

For the PPT tests, the scores for males were as follows: right-hand test scored 12.26 ± 1.98, left-hand test scored 11.91 ± 2.10, two-hand test scored 9.43 ± 1.72, right+left+both scored 33.87 ± 5.59, and assembly test scored 22.48 ± 6.52. For females, the right-hand test scored 13.12 ± 2.44, the left-hand test scored 12.59 ± 2.52, the two-hand test scored 9.98 ± 2.11, right+left+both scored 35.44 ± 6.17, and the assembly test scored 22.49 ± 7.08. According to the standard administration manual of the PPT, the norms are divided at the age of 35 years, with separate standards for males and females both above and below 35 years of age. Our participants fell between 0.14 and 1.89 standard deviations below the norm.

For the MMDT test, the time taken for males was as follows: the dominant hand placement test was 163.59 ± 27.94 seconds, the two-hand turning test was 159.15 ± 35.28 seconds, the preferred hand moving test was 123.85 ± 25.37 seconds, the one-hand turning and placement test was 195.44 ± 40.23 seconds, and two-hand turning and placement test was 118.26 ± 27.11 seconds. For females, the dominant hand placement test was 166.81 ± 27.22 seconds, two-hand turning test was 159.03 ± 29.82 seconds, the preferred hand moving test was 128.33 ± 23.61 seconds, one-hand turning and placement test was 204.25 ± 40.36 seconds, and two-hand turning and placement test was 129.17 ± 32.73 seconds. According to the administration manual, norms are distinguished only by different subtest items and are not established based on age or sex. Comparing our participants’ performance to norms, for both men and women, their performance in all subtests was very poor, with percentile ranks below 1%. The detailed results are summarized in **Table 2**.

**Table 2:** Manual dexterity performance and norms comparison (mean (SD)).

Items	Gender			
	Male (n=34)	Compared to norms	Females (n=36)	Compared to norms
BBT				
Dominant hand	54.44 (9.64)	Below 1.49~4.27 SD	56.61 (9.98)	Below 1.34~2.87 SD
Non-dominant hand	51.15 (10.51)	Below 0.92~4.90 SD	52.92 (8.09)	Below 1.76~3.44 SD
PPT				
Right hand	12.26 (1.98)	Below 0.40~1.83 SD	13.12 (2.44)	Below 0.40~0.61 SD
Left Hand	11.91 (2.10)	Below 0.02~0.49 SD	12.59 (2.52)	Below 0.15~0.63 SD
Both hand	9.43 (1.72)	Below 0.35~0.57 SD	9.98 (2.11)	Below 0.99 SD
Right+Left+Both	33.87 (5.59)	Below 0.15~1.62 SD	35.44 (6.17)	Below 0.14~0.46 SD
Assembly	22.48 (6.52)	Below 0.30~1.65 SD	22.49 (7.08)	Below 1.64~1.89 SD
MMDT (seconds)				
Placing	163.59 (27.94)	Less than one percentile	166.81 (27.22)	Less than one percentile
Turning	159.15 (35.28)		159.03 (29.82)	
isplacing	123.85 (25.37)		128.33 (23.61)	
One-hand placing and turning	195.44 (40.23)		204.25 (40.30)	
Two-hand placing and turning	118.26 (27.11)		129.17 (32.73)	

BBT, Box and Block Test; PPT, Purdue Pegboard Test; MMDT, Minnesota Manual Dexterity Test; SD, standard deviation.

### 3.3 Pearson correlation coefficients among three manual dexterity tests

The BBT showed a significantly strong correlation with PPT for all subtests, with Pearson correlation coefficients ranging between 0.60 and 0.67 (all *p* < 0.001). These findings indicate a robust correlation between the BBT and PPT. Additionally, moderate to strong significant negative correlations were observed between the BBT and MMDT, except for the two-handed turning subtest for the MMDT. The Pearson correlation coefficients ranged between -0.56 and -0.69 (all *p* < 0.001). Regarding the correlations between BBT and the two-hand turning subtest, the Pearson correlation coefficients ranged between -0.48 and -0.52 (all *p* < 0.001), indicating a moderately significant negative correlation between the two. The detailed results are presented in **Table 3**.

**Table 3:** Pearson correlation coefficients among three manual dexterity tests.

Items	BBT			
	Dominant hand test		Non-dominant hand test	
	*r*	*p value*	*r*	*p value*
PPT				
Right hand	0.62	*p* < 0.001	0.59	*p* < 0.001
Left hand	0.60	*p* < 0.001	0.67	*p* < 0.001
Both hands	0.61	*p* < 0.001	0.62	*p* < 0.001
Right+left+both hands	0.67	*p* < 0.001	0.69	*p* < 0.001
Assembly	0.61	*p* < 0.001	0.65	*p* < 0.001
MMDT				
Placing	-0.65	*p* < 0.001	-0.68	*p* < 0.001
Turning	-0.59	*p* < 0.001	-0.67	*p* < 0.001
Displacing	-0.65	*p* < 0.001	-0.65	*p* < 0.001
One-hand placing and turning	-0.56	*p* < 0.001	-0.60	*p* < 0.001
Two-hand placing and turning	-0.48	*p* < 0.001	-0.52	*p* < 0.001

BBT, Box and Block Test; PPT, Purdue Pegboard Test; MMDT, Minnesota Manual Dexterity Test.

### 3.4 Reliability of the BBT

A reliability analysis was conducted using a two-way random model with absolute agreement to assess test-retest reliability. The results indicated that the ICC for the dominant hand test of the BBT was 0.71 (CI: 0.48~0.84; *p* < 0.001), and the ICC for the non-dominant hand test was 0.70 (CI: 0.39~0.84; *p* < 0.001). Taken together, the ICCs for the BBT indicated good intra-rater, inter-rater, and test-retest reliabilities.

### 3.5 Relationship between BBT scores and disease-related factors

A correlational analysis was performed between BBT scores and disease-related factors, including age, disease duration, medication dosage, and severity of psychiatric symptoms (BPRS-C). No significant correlation was found between BBT scores and age, disease duration, or antipsychotic dose (all *p* > 0.05).

Regarding the severity of psychiatric symptoms, the result showed that BBT scores were significantly correlated with the total score and subscales of the BRPS-C, except for the affective subscale. There was a significant moderate correlation between the BBT and total BPRS-C score (dominant hand: r = -0.45, *p* < 0.001; non-dominant hand: r = -0.42, *p* < 0.001) and BBT and BPRS-C-positive (dominant hand: r = -0.54, *p* < 0.001; non-dominant hand: r = -0.46, *p* < 0.001). For BPRS-C activation, the results showed a significant weak-to-moderate correlation (dominant hand: r = -0.39, *p* = 0.001; non-dominant hand: r = -0.30, *p* = 0.012). BPRS-C-negative showed a significantly weak correlation with BBT (dominant hand: r = -0.27, *p* = 0.025; non-dominant hand: r = -0.25, *p* = 0.036). The detailed results are presented in **Table 4**.

**Table 4:** Correlation between BBT scores and disease-related factors.

Items	BBT			
	Dominant hand test		Non-dominant hand test	
	*r*	*p value*	*r*	*p value*
**Age**	-0.02	0.87	0.02	0.89
**Disease duration**	0.01	0.95	0.05	0.69
**Antipsychotics Dosage**	-0.16	0.19	-0.08	0.54
**BPRS-C**				
Total score	-0.45**	< .001	-0.42**	< .001
Positive symptoms	-0.54**	< .001	-0.46**	< .001
Negative symptoms	-0.27*	0.03	-0.25*	0.04
Affection symptoms	-0.11	0.38	-0.17	0.16
Activation symptoms	-0.39*	0.001	-0.30*	0.01

BBT, Box and Block Test; BPRS-C, Chinese version of Brief Psychiatric Rating Scale

**P <0.001; *P<0.05.

## 4 Discussion

This is the first study to explore the validity and reliability of the BBT in individuals with schizophrenia spectrum disorders. Our results showed that manual dexterity performance as assessed by the BBT, PPT, and MMDT was below average in individuals with schizophrenia spectrum disorders, suggesting that they had prominent deficits in manual dexterity. BBT showed a significantly strong correlation with PPT for all subtests and demonstrated moderate-to-strong significant correlations with MMDT, except for the two-handed turning subtest for MMDT. The ICCs for the BBT indicated good intra-, inter-rater, and test-retest reliabilities. The current findings indicate that the BBT has good validity and reliability in individuals with schizophrenia.

Classifying individuals into clearly defined functional groups is critical for optimizing rehabilitation strategies, including therapy selection, dosage, and intensity (39). However, no single assessment tool alone captures the full range of upper limb function in individuals with schizophrenia. This limitation may explain the absence of a universally adopted hand function test in both research and clinical practice for this population. Considering the contribution of range of motion, the BBT offers a simpler, standardized alternative that minimizes measurement error and assessor bias, while placing lower demands on eye-hand coordination. Nevertheless, the PPT and MMDT are capable of assessing in-hand manipulation, bilateral coordination, as well as turning and placing skills. These abilities are crucial for vocational rehabilitation, and both the PPT and MMDT demonstrate greater ecological validity, as they more accurately represent the manual tasks encountered in real-world work environments. This highlights a key limitation of the BBT (27, 40).

Although the BBT is less representative of real-world vocational tasks than the PPT and MMDT, its practicality makes it an appealing choice in clinical settings. Compared to the more complex and time-consuming PPT and MMDT, which consist of at least four subtests each and require a significantly longer time to administer, BBT stands out for its simplicity and efficiency. The BBT, requiring only about 5–10 minutes to complete, involves just two subtests with a single trial each, contributing to a lack of frustration or impatience among participants. In contrast, the PPT demands roughly 15–20 minutes and involves each subtest being tested three times, whereas the MMDT extends the process to 30–40 minutes with two trials and one practice session for each subtest. These more elaborate assessments not only demand greater cognitive effort but also tend to induce negative emotions in participants, potentially affecting manual dexterity performance. Thus, BBT has emerged as a more favorable option for clinical assessment, avoiding the limitations that hamper PPT and MMDT.

Contrary to previous research suggesting a decline in dexterity with age in healthy adults, our findings showed no such correlation, even though the average age of our participants was around 47 years, and their performance on the BBT was below the norm at least 0.7SD, indicating pervasive dexterity deficits across all age groups. We speculate that the likely reason for this is that individuals with schizophrenia exhibit a significant decline in hand dexterity due to the disease itself, and this decline may be more pronounced than that caused by aging. Previous research has shown that diminished manual dexterity is a significant trait of schizophrenia, potentially unrelated to cognitive abilities and possibly not associated with antipsychotic medication (4, 20).

The current study did not find a significant correlation between BBT scores and medication dosage, which is inconsistent with the previous literature. A previous study suggested that motor impairments in individuals with schizophrenia are due to treatment with antipsychotic drugs, which can induce extrapyramidal symptoms and Parkinsonism-like side effects (6). However, other researchers have investigated individuals with schizophrenia who have not been treated with medication and found that motor impairments persist. Therefore, they argued that motor disabilities were related to the disease itself and could not be solely explained by the dosage of the medication (41).

In this study, a notable moderate negative correlation was observed between manual dexterity performance and both the total score and the positive symptom subscale of the BPRS-C, suggesting that greater severity of overall psychiatric symptoms, or specifically in positive symptoms, leads to reduced manual dexterity. Additionally, a weak negative correlation was identified between BBT scores and the negative symptoms subscale of the BPRS-C, indicating a less pronounced effect of negative symptoms on manual dexterity. These findings are similar to those of previous studies, such as Hidese et al. (2018), who found a significant association between more severe negative symptoms and diminished dexterity performance using the PPT and PANSS (42). Similarly, Zakzanis et al. (1998) reported a strong correlation between manual dexterity, as measured using the Grooved Pegboard Test, and negative symptoms, while observing only a weak correlation with positive symptoms using the BPRS (43). The discrepancies between these results and ours might stem from methodological differences. This study assessed correlations directly between subscale scores and dexterity performance, whereas previous studies classified individuals with schizophrenia spectrum disorders based on their predominant symptomology (positive or negative) and examined their correlation with dexterity.

### 4.1 Limitations

This study provides valuable insights while acknowledging the need for a more diverse participant base to enhance our understanding of schizophrenia across different stages. Future research should enrich these findings by including individuals from various settings and with different disease severities. Secondly, all participants were taking antipsychotic medication, which restricted our ability to offer concrete evidence of the impact of antipsychotic medication on dexterity scores. However, substantial evidence and findings from additional studies have challenged this premise. Finally, there may be other manual dexterity-related factors not explored in this study, such as finger strength, suggesting the inclusion of detailed hand measurements in subsequent studies for a comprehensive analysis.

## 5 Implications for occupational therapy practice

The goal of community psychiatric rehabilitation, which emphasizes vocational skills and activities of daily living, highlights the critical role of manual dexterity. This research demonstrates that the BBT, a simple and quick tool, enables occupational therapists to effectively assess manual dexterity in individuals with schizophrenia. The results can serve as a valuable initial reference for comparing a client’s performance against normative data. This information supports appropriate instrument selection in both clinical practice and research, aligned with the specific purpose of the assessment. Subsequently, more comprehensive dexterity assessments that better reflect real-world functional demands can be applied. This approach enables clinicians to accurately evaluate performance, tailor rehabilitation interventions accordingly, and monitor clients’ functional progress more effectively.

## 6 Conclusion

The current findings indicate that the BBT exhibits good validity and reliability for individuals with schizophrenia spectrum disorders. In comparison to the norms for the PPT and MRMT, the BBT norms can be aligned with specific age ranges and sexes for average values and standard deviations. This alignment enables a more accurate reflection and differentiation of individual performance. Therefore, utilizing the BBT to assess the manual dexterity of individuals with schizophrenia not only allows for a simple and rapid assessment but also provides normative data for comparison. This facilitates the development of personalized rehabilitation plans and supports clinical practice in community psychiatric rehabilitation psychiatric rehabilitation.

## Data Availability

The raw data supporting the conclusions of this article will be made available by the authors, without undue reservation.
